# The Gut-Brain Axis in Inflammatory Bowel Disease—Current and Future Perspectives

**DOI:** 10.3390/ijms22168870

**Published:** 2021-08-18

**Authors:** Claudia Günther, Veit Rothhammer, Marisa Karow, Markus Neurath, Beate Winner

**Affiliations:** 1 Department of Medicine 1, Friedrich-Alexander-University Erlangen-Nürnberg, 91054 Erlangen, Germany; Markus.Neurath@uk-erlangen.de; 2 Department of Neurology, Friedrich-Alexander-University Erlangen-Nürnberg, 91054 Erlangen, Germany; veit.rothhammer@uk-erlangen.de; 3 Institute of Biochemistry, Friedrich-Alexander-University Erlangen-Nürnberg, 91054 Erlangen, Germany; marisa.karow@fau.de; 4 Department of Stem Cell Biology, Friedrich-Alexander-University Erlangen-Nürnberg, 91054 Erlangen, Germany

**Keywords:** gut-brain axis, IBD, MS, PD, ex vivo organ models

## Abstract

The gut–brain axis is a bidirectional communication system driven by neural, hormonal, metabolic, immunological, and microbial signals. Signaling events from the gut can modulate brain function and recent evidence suggests that the gut–brain axis may play a pivotal role in linking gastrointestinal and neurological diseases. Accordingly, accumulating evidence has suggested a link between inflammatory bowel diseases (IBDs) and neurodegenerative, as well as neuroinflammatory diseases. In this context, clinical, epidemiological and experimental data have demonstrated that IBD predisposes a person to pathologies of the central nervous system (CNS). Likewise, a number of neurological disorders are associated with changes in the intestinal environment, which are indicative for disease-mediated gut–brain inter-organ communication. Although this axis was identified more than 20 years ago, the sequence of events and underlying molecular mechanisms are poorly defined. The emergence of precision medicine has uncovered the need to take into account non-intestinal symptoms in the context of IBD that could offer the opportunity to tailor therapies to individual patients. The aim of this review is to highlight recent findings supporting the clinical and biological link between the gut and brain, as well as its clinical significance for IBD as well as neurodegeneration and neuroinflammation. Finally, we focus on novel human-specific preclinical models that will help uncover disease mechanisms to better understand and modulate the function of this complex system.

## 1. Introduction

Inflammatory Bowel Diseases (IBDs) are prototypic immune-mediated inflammatory diseases (IMIDs) that affect the gastrointestinal (GI) tract and have a globally increasing prevalence and multifactorial etiology. The two main entities include Crohn’s Disease (CD) and ulcerative colitis (UC), which differ in extent and localization of inflammation. Previous clinical and preclinical studies have highlighted important components in the pathophysiology of both diseases. These include (a) local immune-cell populations and their associated mediators [1,2,3], (b) genetic predisposition [4,5,6], (c) the epithelial barrier [7,8,9] as a shield of the bowel wall against (d) altered intestinal microbiota, as well as other environmental factors that can be altered, for example, via diet [10,11,12,13]. Emerging evidence further indicates that the enteric nervous system (ENS) strongly influences mucosal immunity and thus might represent an important contributor to IBD development and progression [14,15]. Disruption of ENS function may have bidirectional consequences for the gut and the central nervous system (CNS) [16]. In this context, visceral hypersensitivity and chronic pain are common debilitating symptoms of IBD suggesting an interaction between the gut, the ENS and CNS. It is currently believed that pain, including neuro-immune interactions in the inflamed gut, mediate an increase in intestinal permeability that ultimately leads to an aggravation of disease activity and that resolution of inflammation is associated with reduced abdominal pain [15,17]. Interestingly, UC was originally considered a psychosomatic disorder until the latter half of the 20th century [18]. With the discovery in 1954 of the therapeutic effect of steroids for UC [19,20], it became obvious that it originates in the digestive tract and is a primary severe chronic inflammatory disease. In contrast, irritable bowel syndrome (IBS) is a prevalent functional gastrointestinal disorder characterized by chronic abdominal pain or discomfort and altered bowel habits due to visceral hypersensitivity. Chronic, subtle and low-grade subclinical inflammation of the intestinal mucosa may also be involved in the pathology of this disease condition (reviewed in Ng et al., J Inflamm Res) [21]. Moreover, intestinal malabsorption syndromes (e.g., Vit B12 deficiency) can cause severe neurologic and neuropsychiatric symptoms culminating, e.g., in funicular myelosis or psychosis [22].

**Figure 1:**
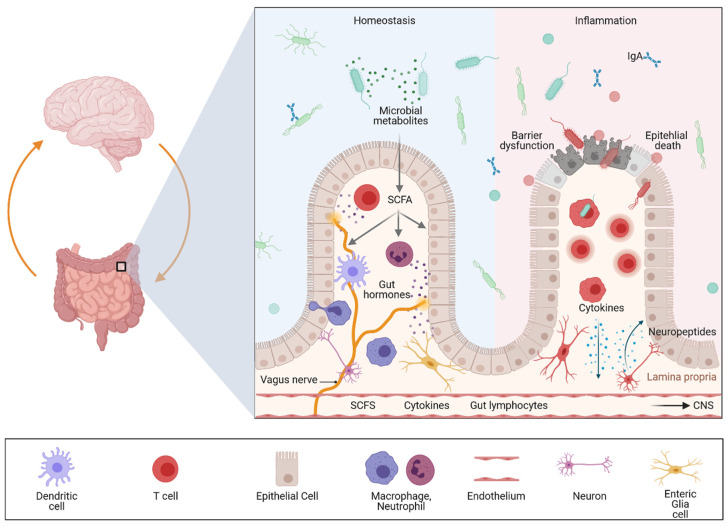
Impact of intestinal inflammation on gut–brain communication. The figure was generated with biorender.com (8 July 2021). Multiple pathways exist through which gut–brain communication can be modulated including neural, hormonal, metabolic, immunological, and microbial signals. Signaling events from the gut can modulate brain function, and recent evidence suggests that a dysregulated gut–brain axis plays a pivotal role in linking gastrointestinal and neurological diseases involving neuroinflammation as well as neurodegeneration. Cytokines released by the mucosal immune system in response to local inflammation or infection can be released in the periphery reaching the CNS via the bloodstream. Similarly, circulating gut-primed immune cells can cross the blood–brain barrier (BBB) and modulate immune responses in the CNS. In addition, bacterial metabolites, such as short-chain fatty acids (SCFAs) are neuroactive metabolites of dietary fibers that can further influence gut–brain communication and neuroinflammation. In addition to immune signals and microbial metabolites, signaling from the vagus and enteric nervous system affect gut mucosal and anatomically distant tissues such as the CNS. These peripheral signals promote neuroinflammation and neurodegeneration.

The close association between the gut and the brain was uncovered in the 21st century and is now known as the gut–brain axis. Microbial–neuro–immune–endocrine modulation of this bidirectional communication system likely plays a pivotal role in the pathogenesis of gastrointestinal and neurologic diseases (Figure 1).

Accordingly, enteric dysbiosis and translocation of bacterial products as well as inflammatory soluble factors derived from the inflamed intestinal mucosa across the gut–epithelial barrier and blood–brain barrier (BBB) have been widely recognized as major factors for structural and functional alterations in the CNS. Thus, it is not surprising that the impact of mucosal immune dysfunction and the contribution of the gut microbiota to peripheral inflammation is also in the focus of neurodegenerative diseases including synucleinopathies like Parkinson’s disease (PD) [23] and IMIDs such as multiple sclerosis (MS) [24]. Recent population-based studies have shown that patients with IBD have a higher risk for developing PD [25,26,27,28,29]. In general, PD is a neurodegenerative disorder of the ageing brain, resulting in gradual movement impairment including hypokinesia, rigor and resting tremor. It is characterized by progressive loss of dopaminergic neurons in the substantia nigra and the appearance of Lewy bodies (abnormal aggregates of misfolded proteins, including a-synuclein) [30,31]. Epidemiological analyses suggest that patients with IBD are at a 20–90% higher risk of developing PD than individuals without an inflammatory phenotype in the gut.

Similarly, there is a bidirectional association between MS and IBD. Data from meta-analyses derived from 10 studies including more than one million patients (0.08% with comorbidity of IBD and MS) indicate that the cumulative risk ratio for IBD/MS comorbidity was 1.54 (1.53 for the risk of MS in IBD and 1.55 for the risk of IBD in MS patients). In general, data derived from this systematic review of published patient data suggest that both IBD and MS patients seem to have a 50% increased risk of MS or IBD comorbidity, with no difference between CD or UC [32]. Of note, while several epidemiological studies observed a higher incidence of neuroinflammation and neurodegeneration among patients with IBD compared to individuals without IBD, the impact of biologicals such as anti-TNF-α was controversially discussed. Some studies report that anti-TNF-α therapy is contraindicated in patients who have both IBD and MS since exposure to this biological increases the incidence of MS in patients with IBD to 43%. Other studies demonstrated that early exposure to anti-inflammatory anti-TNF therapy was associated with substantially reduced PD incidence [26,33,34].

Importantly, depending on the study population, the risk for developing CNS pathologies among IBD is highly variable and data regarding the individual risk for neurodegeneration among CD und UC patients largely vary. One crucial variable is the age of diagnosis: the incidence of PD in patients with IBD onset over 60 years is higher, mostly because PD is an age-related disease as mentioned above. Often, a variety of drugs is administered to elderly patients, which increases the risk of drug-induced neurological diseases. In summary epidemiological studies clearly demonstrate the risk for PD, MS or other pathologies of the CNS among patients with IBD; however, the exact numbers need to be refined. While the risk of patients with IBD developing extraintestinal manifestation in the CNS is strongly supported by clinical and preclinical studies, data on the incidence of intestinal inflammation among PD patients are scarce and still a topic of considerable debate. This might partially be due to the fact that IBD is usually diagnosed in younger individuals, while neurodegeneration is rare in patients under 50. In contrast, the association between MS and IBD is bi-directional since an increased incidence of both IBD among MS as well as MS among IBD has been observed [32,35,36,37]. MS is primarily an inflammatory autoimmune disorder characterized by targeted destruction of myelin, a major component of the CNS, by autoreactive inflammatory T cells. It is interesting to note, that common MS drugs exert their anti-inflammatory and organ-protective actions based on mechanisms that may also have an impact on the intestine. As such, the a4 integrin targeting monoclonal antibody natalizumab is efficient at blocking VLA-4 (integrin α4β1) mediated entry of autoreactive T cells in the CNS. However, a4 also forms a heterodimer as integrin α4β7, which has been proven relevant for the entry of pro-inflammatory immune cells into the intestinal wall (reviewed in PMID [38,39]). In these lines, modulation of sphingosine-1 phosphate receptors (S1PR) by fingolimod or the newly developed drugs siponimod, ozanimod, and ponesimod, traps lymphocytes within the lymph node and thus limits their entry into the target organs. While this strategy is highly efficient in autoimmune CNS diseases such as MS, it may yield beneficial effects in inflammatory bowel diseases as well. Indeed, several preclinical studies have examined these modes of actions and may provide useful in future clinical approaches [40,41,42]. Neurodegeneration is a frequent consequence of severe neuroinflammation in MS. In patients with IBD demyelinating events in both central and peripheral nervous systems have been observed. Based on a literature search of available clinical studies, it has been speculated that Infliximab and other anti-TNF-α agents might be responsible for drug-induced demyelination in patients with IBD [43]. Another important player along the gut–brain axis might be the brain glymphatic system [44,45]. This paravascular space of the CNS is gaining research attention as immune mediator and for its relevance in age-related changes of brain function, as well as in the pathogenesis of neurodegenerative and neuro-inflammatory diseases. An interaction of this system and involved receptors along the gut–brain axis is likely.

In summary, there is increasing evidence from epidemiological as well as clinical and preclinical studies that demonstrated the impact of gut-to-brain communication and vice versa on the pathogenesis of respective diseases.

## 2. Genetic Evidence for an Association between Gut and Brain in the Context of Inflammation

The majority of PD cases are of sporadic origin; however, about 10% are familial and the most common monogenic forms of PD are pathogenic variants on the *LRRK2* gene that encode leucine-rich repeat kinase 2 (LRRK2) [46], a multidomain protein with a catalytic core that can fulfill kinase and GTPase activity. It also has a scaffold function allowing LRRK2 to interact and recruit several other signaling molecules. The coding variants associated with PD cluster within the enzymatic core of LRRK2 and are thought to disrupt the enzymatic functions of this protein. Accordingly, preclinical studies have indicated that targeting the activity or expression of LRRK2 is neuroprotective. Therefore, small-molecule inhibitors of LRRK2 and antisense oligonucleotides have been developed. Interestingly, variants of this gene are not merely associated with an increased risk of Parkinson’s disease. In the context of PD, it has been demonstrated that LRRK2 deficiency can result in the deregulation of autophagy, and polymorphisms of this gene have been linked to an increased risk of CD [47,48,49,50,51].

Recent genome-wide association studies (GWAS) have shown that the association between the *LRRK2* locus and IBD includes several *LRRK2* genetic variants [49]. Although the interaction of alterations in LRRK2 function and CD mechanisms is still unknown, increasing evidence supports the fact that LRRK2 plays a role in mediating autophagy in Paneth cells, which would explain its strong association with CD because defects in Paneth cell autophagy have been described as hallmarks of this IBD prototype [52]. In line with this notion, preclinical studies using mice with a LRRK2 deficiency showed a specific impairment in the expression of antimicrobial peptides by Paneth cells [53]. Interestingly, a recent hypothesis-driven analysis of 56 single nucleotide polymorphisms (SNPs) associated with CD susceptibility found that *LRRK2* but not *ATG16L1* was associated with Paneth cell defect in Japanese CD patients [54]. However, it is important to mention that, while authors in this study could not find a correlation, the ATG16L1 polymorphism has been linked to Paneth cell dysfunction in many other clinical and preclinical studies and associated with gut microbiota dysbiosis [51,55,56,57,58]. Of note, besides *LRRK2, NOD2* is the most strongly associated risk factor for Paneth cell dysfunction in CD, which was identified 20 years ago [59,60].

Unique variants with incomplete penetrance in *LRRK2* and *GBA* have been shown to be strong risk factors for PD in certain populations. In addition, over 20 common variants with small effect sizes have been shown to modulate the risk for PD [61]. A large Caucasian study identified risk loci at *SNCA* and *MAPT* (encoding microtubule associated protein tau) and provided supporting evidence for the association at *LRRK2* among others [61]. Another link towards inflammation is derived from an additional risk locus at *HLA-DRB5* (major histocompatibility complex class II, DR beta 5) [62]. Just recently a GWAS meta-analysis including PD patients and their first-degree relatives identified 38 novel susceptibility loci, including *NOD2* [63]. As mentioned above *NOD2* is highly associated with IBD. It is important to note that beside *LRRK2* and *NOD2,* several other risk loci are shared between CD and PD including *MROH3P*, *HLA-DRB5*, *CCNY*, *LRRK2*, *MAPT*, *SYMPK*, and *RSPH6A* [49]. In addition, four variants are shared with UC (*GUCY1A3*, *HLA*, *BTNL2*, and *TRIM10*).

The genetic risk factors associated with MS and IBD are not well described in the literature. Genetic studies of MS cohorts suggest that this autoimmune disease is provoked following exposure to environmental factors, which might be responsible for loss of tolerance and peripheral activation of myelin-specific T cells. GWAS have supported the complexity of MS pathology and uncovered immune-related gene variants linking MS to other autoimmune diseases such as IBD [64]. Further systematic studies are needed to better delineate the genetics between IBD and MS.

## 3. Evidence from Gut–Brain Communication in Preclinical Mouse Models

Beside the epidemiological and genetic evidence, several preclinical studies support the importance of gut–brain communication in intestinal inflammation. In the dextran sulfate sodium (DSS)-induced colitis, a rodent model of IBD, it was demonstrated that, parallel to local inflammatory responses in the gut mucosa, increased expression of *IL6* and *iNOS* (Nitric oxide synthase, inducible; *NOS2*) was found in the cerebral cortex. The authors further described microglial activation by increased immunoreactivity for the pan-myeloid cell marker ionized calcium-binding adapter molecule 1 (Iba1) and elevated cytokine levels [65]. Another study analyzed the impact of intestinal inflammation by DSS administration in a model of dopaminergic neurodegeneration by LPS injection in the substantia nigra [66]. The authors demonstrated that inflammatory responses in the gut reinforced the inflammatory and deleterious effects of LPS induced neuroinflammation as indicated by increased levels of TNF-α, GFAP, and IL-6 in serum and the substantia nigra of the animals.

Recently, chronic mild gut inflammation was shown to accelerate brain neuropathology and motor dysfunction in α-synuclein mutant mice [67]. The age of onset of motor dysfunction was significantly earlier in DSS-treated α-synuclein mutant mice compared to mutant mice that were not challenged with DSS. Based on these data, the authors concluded that interventions to reduce gut inflammation might be beneficial for preventing and treating PD. Interestingly, there are several recent animal studies suggesting that α-synuclein, a key protein involved in PD pathology, accumulates not only in the brain but also in the gut. Surprisingly, this could not only be observed in α-synuclein transgenic mice but also in mice subjected to DSS (experimental colitis drives enteric α-synuclein accumulation and Parkinson-like brain pathology). In line with previous examinations, this study observed that experimentally induced colitis in transgenic mice exacerbated α-synuclein pathologies in the CNS. While highly interesting, the effect of α-synuclein aggregates on ENS homeostasis has not been studied. So far, only one study demonstrated that increased α-synuclein expression following colitis was associated with phosphorylation in the myenteric plexus of common marmosets [68].

In summary, available studies suggest that inflammation is associated with peripheral alterations affecting CNS homeostasis through factors accumulating in the gut or systemically. In line with this hypothesis, another recent study demonstrated activation of microglial cells and reduction in occludin and claudin-5 expression in the brain suggesting an impaired BBB following experimental colitis [69]. These data further suggest that DSS-induced colitis increases systemic inflammation which then results in cortical inflammation via up-regulation of serum cytokines. Interestingly, the same group further observed that a decrease in dopaminergic function was associated with an increase in gastrointestinal inflammation, suggesting a bidirectional gut–brain interaction. Accordingly, mice studies showed that Experimental Autoimmune Encephalomyelitis (EAE), a model for MS in rodents, is accompanied by loss of mucosal immune homeostasis [70]. The authors studied intestinal tissue homeostasis following EAE and found significant structural alterations in epithelial morphology accompanied by increasing levels of infiltrating pro-inflammatory T-cells. They could further demonstrate an increased infiltration of pro-inflammatory Th1/Th17 cells and a reduced number of regulatory T cells in the gut during EAE. The adoptive transfer of encephalitogenic T cells, isolated from EAE-diseased animals to healthy mice was sufficient to induce intestinal changes similar to those observed during the onset of EAE. These data suggest that the loss of intestinal homeostasis may support EAE progression by inducing a systemic and chronic inflammatory state.

In addition, several early studies in animal models provided strong evidence that alterations in the composition of the intestinal microbiota are associated with neurological alterations. At the same time, stress can disturb the composition of the gut microbiota. The impact of gut microbiota on neuroinflammation and neurodegeneration will be discussed in more detail below.

## 4. The Different Levels of Gut–Brain Communication

The gut–neuronal communication network is highly complex and involves cell of the innate and adaptive immune system, epithelial and endothelial cells as well as signaling molecules. These include microbial-derived metabolites, immune cell-derived soluble factors such as cytokines, and neuropeptides (neurotransmitter, hormones) released by mucosal cells. Networking takes place locally in the gut via the ENS or by direct interaction with the CNS. Thus, this communication network has to be tightly controlled as disruption at any step along the gut–ENS–CNS axis is involved in the pathogenesis of a diverse range of diseases including immune-mediated neuroinflammatory diseases such as IBD and MS and synucleinopathies, including PD.

### 4.1. Neuronal Communication

The GI tract is the only internal organ that has its own independent nervous system, the enteric nervous system (ENS) [71,72]. This digestive system can be innervated by intrinsic enteric neurons and by extrinsic efferent and afferent nerves. Accordingly, the ENS can be separated into the intrinsic enteric nervous system (iENS), which consists of the myenteric and submucosal plexus containing enteric neurons and glial cells, and the extrinsic sensory nervous system (eENS) comprising the primary afferent and autonomic fibers that provide the communication between the gut and the CNS [73,74,75].

Interestingly, it has been increasingly recognized that the ENS plays a far more complex role than “just” controlling sensorimotor functions or intestinal mucus secretion. Particularly in the last year, there has been growing evidence highlighting a fundamental role of bidirectional communication between the mucosal immune system and the neuronal network concerning intestinal inflammation. This neuro-immune crosstalk can be influenced by additional signals such as soluble factors released by the intestinal microbiota or intestinal epithelial cells under steady-state conditions but also in the context of pathologies. Accordingly, the concept of neurogenic inflammation was established.

It has been shown that neuropeptide-containing (peptidergic) neurons within the colonic wall are key players in neurogenic inflammation as they release neuropeptides into the adjacent tissue. These peptides can induce vasodilation, plasma extravasation and leukocyte migration. Moreover, these neuropeptides have been shown to not only regulate intestinal homeostasis but also inflammation [76]. Accordingly, experimental studies could demonstrate that peptidergic neurons release neuropeptides that orchestrate colonic inflammation in a complex way. Calcitonin gene-related peptide (CGRP) and substance P (SP) seem to be the link between neuronal activation and the consecutive mucosal immune response. In murine colitis models, mice deficient in neutral endopeptidase (an enzyme responsible for the extracellular degradation of SP) displayed and aggravated colitis, while mice deficient in substance P showed a strong attenuation of colitis severity [15,74,77]. In sharp contrast, CGRP-deficient mice showed increased susceptibility to experimental colitis. Neuropeptide release is controlled by transient receptor potential (TRP) channels; therefore, neuropeptides released locally in the gut may function as mediators at the interface between the nervous system, the mucosal immune system and other cell compartments such as the epithelium or endothelium. Interestingly, recent single cell analyses revealed a significant expression of risk genes for diseases that feature intestinal and CNS involvement in the ENS, suggesting that it is involved in gut–brain disease communication [16].

### 4.2. Microbial Communication

While the importance of the gut microbiome was described some time ago for IBD and a variety of other immune and metabolically driven diseases, the essential role of the gut microbiota in CNS inflammation was discovered only a few years ago [78]. To understand why environmental factors derived from the gut microbiota have such a huge impact on host physiology and pathophysiology, it is important to mention that the gastrointestinal tract harbors more than 100 trillion microbial cells belonging to more than 1000 bacterial species [79]. This results in 10 times more microbial than human cells in our body. Accordingly, it is not surprising that the microbiome plays an important role in the pathogenesis of intestinal and extraintestinal diseases. In line with this, multiple lines of evidence have recently emerged on the role of the gut microbiota in neuroinflammation and neurodegeneration.

Several clinical studies highlighted a reduced diversity and altered composition of the gut microbiota (dysbiosis) not only in mouse models of neuroinflammation and neurodegeneration, but also as a common feature of patients with PD [23,80,81,82,83] and MS [84,85,86,87,88,89,90,91]. While dysbiosis has been shown in many clinical and preclinical studies, a disease-relevant microbiota for neuroinflammation or neurodegeneration is debatable. In addition, it still remains unclear whether dysbiosis can modulate inflammatory processes in the CNS or if it is merely the consequence of neuroinflammation/neurodegeneration. In support of a rather causative function of gut microbes, germ-free mice were resistant to spontaneous EAE, a striking notion that was explained by a lack of local activation of T cells in the gut and the subsequent deficient triggering of pathogenic antibody production by activated B cells. A translational study could demonstrate that transplanting faecal microbiota from PD patients exacerbated motor dysfunction in an α-synuclein transgenic mouse model [92]. Another recent study further demonstrated the huge impact of the intestinal microbiota in a genetic mouse model mimicking PD pathology. Although pathologic variants in PINK1 and PARKIN in patients with PD lead to disease progression with nearly 100% penetrance, *Pink1*-knockout mice do not develop PD-like symptoms [93]. However infection of these mice, during early life, with a Gram-negative bacterium (*Citrobacter rodentium*) that causes mild colitis, was sufficient to trigger PD-like symptoms later in life [94]. Mechanistically, it has been suggested that the dysbiotic microbiota, associated with alterations in microbial metabolites, may induce neurodegeneration in PD by influencing gut barrier function. Increased leakiness would further favor an inflammatory environment and oxidative stress that might promote α-synuclein accumulation in the ENS. This might further be propagated via a prion-like mechanism across the vagal nerve to the CNS [95]. Experimental support for this hypothesis has recently been demonstrated in animal models of neurodegeneration [96,97]. Mechanistic studies revealed that communication between gut microbes and the ENS can shape Th17 responses, leading to increased inflammation in the CNS [98,99,100,101]. Discoveries on CNS pathophysiology provide functional evidence on how the gut microbiota influences immune responses not only locally in the gut but also in the CNS via bacterial-derived metabolites, such as short-chain fatty acids (SCFA). Accordingly, oral administration of specific microbial metabolites to germ-free mice was sufficient to trigger neuroinflammation, motor impairments and α-synuclein pathology [92,102]. It is important to mention that, in the context of EAE, metabolites of dietary tryptophan produced by the commensal microbiota limit CNS inflammation and neurodegeneration by influencing maturation and function of microglia and astrocytes [24,103]. Importantly, tryptophan can be metabolized by the gut microbiota and the host [104]. For example, it can be sensed by enteroendocrine cells, which results in the production of serotonin (5-HT), a neurotransmitter critically involved in the modulation of the ENS (enteric neuron) and CNS [105,106,107]. Beside this immune-modulatory function, the gut microbiota can modify drug bioavailability and efficacy. For example, it has been demonstrated that the efficacy of Levodopa (L-dopa), the major drug treatment for Parkinson’s disease, is hugely variable among individuals, depending on the composition of their microbiota [108]. For the sake of completeness, evidence from mouse models further suggests that the gut microbiota may indirectly influence the CNS directly via the vagus nerve route [109].

It is well known that the interplay between genetic predisposition, the gut microbiota and environmental influences results in a dysregulated intestinal immune response that initiates and perpetuates the mucosal inflammatory reaction [110]. Therefore, in the next chapter we summarize some of the most important immunological factors linking gastrointestinal inflammation with pathologies of the CNS.

### 4.3. Immunological Cross Talk

The role of the immune system in IBD is well established. Accordingly, immune-based therapies and diagnostics have proven their enormous potential in the treatment of IBD and are currently revolutionizing medical practice across numerous disciplines [1,111]. As mentioned above, mucosal inflammation has been implicated as an essential driver of neuroinflammation and neurodegeneration. For example peripheral IL-17-producing T cells have been shown to induce neuronal death in a PD-related human autologous stem-cell-based model. These preclinical studies identified IL-17-producing T cells as novel key drivers of PD associated neurodegeneration [99,112]. Emerging evidence suggests that these T cells might be primed in the gut and recruited to the brain. In line with this hypothesis, the gut microbiome and microbial metabolites contributed to the commitment of mucosal T cells towards a Th17 phenotype that exacerbated neuroinflammation in EAE [113]. Recent advances in an ex vivo organ culture system further demonstrated that the gut harbors immune-modulating microbes that activate neurons locally in the gut by influencing neuronal gene expression via mucosal Th17 cells that then exacerbate inflammation in the CNS, highlighting the mucosal immune system as the master regulator of gut–brain communications [114,115,116]. However, the exact pathways and antigens sensed by gut antigen-presenting cells are still unknown. Moreover, it is not clear if their recruitment to the CNS is the result of a “misguided” instructive process. An alternative hypothesis suggests that gut-derived “environmental” cues being released into the systemic circulation due to impaired barrier function can directly influence neuroinflammation in the brain by engaging surface pattern recognition receptors on resident cells or ligand-driven transcription factors such as the aryl hydrocarbon receptor (AHR) system [103]. Beside these environmental factors, pro-inflammatory cytokines such as IL-1β, IL-6, and TNF-α, that are present in the inflamed gut, are released into the circulation, leading to systemic inflammation affecting the brain. Accordingly, elevated levels of circulating inflammatory cytokines are associated with neurodegeneration and neuropsychiatric disorders in humans. In this context it has been demonstrated that effective anti-inflammatory therapy by cytokine blocking agents reduced the pain perception in the brain in patients suffering from IBD [17]. The gut also releases cytokines involved in local neuron–immune cross-talk. For example, the alarmin cytokine IL-33, which is released upon epithelial cell damage, promotes the production of serotonin by enteroendocrine cells. IL-33-mediated serotonin release activated enteric neurons, which subsequently promoted gut motility [117]. Interestingly, stimulation of Group 2 innate lymphoid cells (ILC2s) by IL-33 treatment or following *Nippostrongylus brasiliensis* infection resulted in the upregulation of the transcript for *tryptophan hydroxylase 1* (Tph1), the rate-limiting enzyme in serotonin biosynthesis [118].

In summary, there is growing evidence suggesting that the gut is strongly involved in various neurological diseases via direct and indirect mechanisms. The key components are intestinal microbes and their products (e.g., metabolites) and immune education in the mucosal immune system, including immune cells releasing proinflammatory cytokines. Key to the regulation of these processes is the intestinal epithelium, which is capable of translating microbial and inflammatory signals to the immune system and secreting peptides as well as hormones, which are involved in the metabolic processing of dietary nutrients. Although this network is of strong clinical relevance for both intestinal and neurological diseases, we are just beginning to understand the underlying molecular mechanism and how organ crosstalk is regulated during health and disease. Accordingly, we need novel model systems to better understand microbiota–gut–brain communication on a cellular level. In the last chapter we therefore focus on novel human-specific preclinical model systems that will help to uncover disease mechanisms, which might allow us to better understand and modulate the function of this complex system.

## 5. Ex-Vivo Organ Models

Organoids are miniature three-dimensional assemblies of cells containing structures that mimic the architecture of the respective organ. The fascinating aspect is the intrinsic ability of cell aggregates to self-organize. Specifically, they follow the basic intrinsic patterning events comparable to those in place during natural organ development. This makes them a useful tool, both for the investigation of developmental organogenesis as well as for disease modeling.

The building block for organoids are stem cells. Two different techniques are used: one involves adult tissue resident stem cells (ACS) derived from the respective organ where they support regenerative mechanisms on site; the other source is pluripotent stem cells (PSC), which can develop in almost any cell type of the human body. PSCs can be derived from either embryonic stem cells (ES cells) or induced PSCs (iPS cells). The latter are obtained by reprogramming of somatic cells using a set of 4 transcription factors (known as Yamanaka factors) [119].

### 5.1. Brain Organoids

The complex process of human brain development is achieved through the spatially and temporally regulated release of key patterning factors. Initially, the goal of in vitro modeling of brain cells was achieved by PSC derived two-dimensional neural cultures. Compared to the brain, the complexity of these cultures is low, and from the beginning the driving motive was for more advanced in vitro models concerning cell composition, maturation, and tissue architecture. The ability of PSC cells to form rosette-like structures, delineating the self-organization potential of neural progenitors to form neural tube-like structures was found two decades ago [120] and then refined, resulting in structures reminiscent of early stages of brain development [121,122,123]. A major breakthrough came with a series of publications from the Sasai and Knoblich labs [124,125]. Inspired by the cell’s intrinsic development program and self-patterning ability, they provided a pea-size brain model, termed “brain organoid”. Specifically, this work demonstrated the generation of broad brain regional identities with a minimal set of chemical guidance. The Lancaster study implemented two new crucial experimental steps: (I) embedding of pre differentiated cellular aggregates in matrigel, followed by (II) spinning in a three-dimensional device over the course of months. These experimental changes led to the reorganization and expansion of the neuroepithelium. Brain organoids contained cortical regions but also generated a large variety of brain regions including the midbrain, hindbrain and retina.

The ultimate comparison of the complexity of this model is the human brain. Here, OMICS techniques like single-cell RNA-Sequencing (scRNA-seq) were instrumental. Analyses of brain organoids at different times of differentiation showed that the developmental steps and fates of cell populations mimicked processes in the human fetal brain [126]. The latest updates on the generation of brain organoids provided 3D structures with brain-specific regional identities, including the forebrain [127], midbrain [128] and hippocampus [129]. As further outlined below, the ability to introduce epithelial barriers into organoid models is highly relevant when considering organoids for studying inter-organ communication. In a recent study, such a selective barrier was successfully modeled for the first time, namely, through choroid plexus organoids, including self-contained compartments [130].

Moreover, the fusion of regionalized brain organoids indicated that brain circuits can be modeled in these assembloids [131,132]. Another important aspect of using brain organoids for modeling concerns the reconstruction of circuits of interest. A brilliant example is the reconstruction of the motor circuit using 3D cortical motor assembloids [133].

### 5.2. Enteric Nervous System (ENS)

Human stem cell-based modeling of the enteric nervous system (ENS) is less frequently found than differentiations of CNS neural cells. The ENS, a vast network of neuronal and glial cells, is derived from neural crest (NC) progenitor cells. Studer and Fattahi implemented human PSC-based protocols for NC induction and regional specification [134,135], which led to the development of a robust method for directing the fate of hPSCs towards the enteric NC and further to the vagal ENS. The application of hPSC-derived enteric neural lineages provided a powerful platform not only for ENS-related disease modeling of neurodevelopmental processes like Hirschsprung disease [135] but also for studies of cell–cell interaction with CNS-derived neural cells and with the intestinal system to better understand inflammatory diseases.

### 5.3. Intestinal Organoids

The human GI tract can be separated into the foregut, midgut, and hindgut [136,137]. Each of these regions gives rise to defined tissues and organs. The foregut endoderm is the embryonic progenitor for the oral cavity, pharynx, esophagus, stomach, proximal duodenum and parts of the hepatobiliary system such as the liver parenchyma and pancreas. Other parts of the small intestine (distal duodenum, jejunum, ileum) as well as colon, rectum, anal canal, and also the epithelium of the bladder and urethra develop from the midgut and hindgut endoderm. Organoids recapitulating the GI tract or hepatobiliary–pancreatic (HBP) system can be derived from PSC and adult stem cells. Following the direction of PSC in an endodermal direction, individual protocols mimic the respective developmental cues (reviewed by [138,139,140]). Intestinal organoid protocols are based on modulation by *WNT3A*, *Notch*, *FGF4*, *EGF*, and *BMP/Nodal* signaling [141,142,143]. A detailed protocol for generating 3D human intestinal tissues (organoids) in vitro from human PSC was provided by the Spence lab [144]. Recent work by the Helmrath and Wells groups introduced a tissue-engineering approach with PSCs to generate human intestinal tissue containing a functional ENS [145]. ENS-containing intestinal organoids grown in vivo formed neuroglial structures similar to a myenteric and submucosal plexus, contained functional interstitial cells of Cajal, and showed electromechanical coupling that regulated waves of propagating contraction. The authors further showed an example of how this system can be used to study motility disorders of the human gastrointestinal tract. Interestingly a recent study demonstrated that patient-derived (UC) organoids recapitulated colitic reactivity offering opportunities to tailor interventions to the individual patient [146]. In this context, a recent study demonstrated that PSC-derived intestinal organoids can be used for compound testing [147].

Beside PSC-derived organoids, intestinal as well as biliary and pancreatic organoids can easily be derived from the tissue of the adult stem-cell niche or adult somatic tissue. When these stem cells are grown in a three-dimensional environment, they self-organize into organoids that replicate key structural and functional features of the corresponding part of the GI or HBP tract they are derived from. Interestingly, even they share a common developmental origin, the physical turnover is highly variable between the different organs, which results in the fact that the efficiency of generating the organoids and maintaining the cultures varies among the organs [148,149,150]. Accordingly, these adult stem cell-derived organoids can be differentiated from organoids derived from high-turnover stem cells (e.g., intestinal epithelium) and slow-turnover organs such as the liver and pancreas [151,152]. The gastrointestinal tract has a further advantage as its maintenance and repair relies on a small population of actively cycling tissue-resident stem cells found in a very specific location at the bottom of the invaginations of the mucosa known as “crypts” [148]. In sharp contrast, the liver and pancreas do not contain a defined stem-cell niche, making it difficult to obtain the corresponding progenitor cells. Of note, small intestinal organoids derived from adult stem cells of the gut were the first 3D miniorgans described more than 10 years ago by the group of Hans Clevers [153]. He showed that two factors were important for maintaining these cells in a 3D structure: (I) an extracellular matrix (mostly with matrigel, a solubilized basement membrane preparation extracted from the Engelbreth–Holm–Swarm mouse sarcoma) and (II) a culture medium supplemented with growth factors that resemble the in vivo stem-cell niche environment. This medium mainly includes factors that stimulate the WNT signaling pathway and alters BMP signaling. Intestinal organoids can be used to study the pathophysiology of a variety of human inflammatory diseases, such as IBD and celiac disease, and infectious disorders (*Helicobacter*, *Salmonella*) as well as metabolic, and neoplastic diseases [7,154,155,156,157,158,159,160,161,162,163]. Patient-derived organoids further allow drug screening and personalized approaches for treating diseases. Importantly, in sharp contrast to those of the brain, intestinal organoids, including epithelial and ENS organoids, can be derived from PSC and ASC. This allows the comparison of organoids with identical genetic background, but only one population (ASC) was exposed to an inflammatory environment.

## 6. Modeling the Gut–Brain Axis or Barriers in Human Cell-Based Systems

While to our knowledge no robust gut–brain axis model has been described, combining the two systems experimentally might be a major step towards a better understanding of bidirectional gut–brain communication. In this regard, implementing experimental approaches to study barrier functions is of major importance. Here, the mucosal surface of the intestine and the blood–brain barrier are particularly relevant. Addressing these barriers is essential for (1) identifying and characterizing key mechanisms of gut–brain interaction, (2) defining the temporal regulation of these pathways in IBD and neurodegeneration and neuroinflammation, and (3) evaluating their functional impact and their therapeutic potential. As a prerequisite for generating epithelial barriers in organoids, the major cellular contributors, endothelial cells, have to be implemented. The first reports on the endothelialization of brain organoids showed encouraging results on the general feasibility of this goal. Importantly, following transplantation of such organoids using mice as a host, vascularization through host–graft interactions was shown [164,165,166].

Refinements will be needed to define the conditioned media of one organ fitting another, and the respective effects within the other system will need to be carefully tested. Alternatively, co-cultures will be an exciting tool for answering questions of direct cell–cell interaction. Critical and well-controlled steps will include the combination of different growth factors and adapting the respective timelines since the proliferative potential of the intestinal system exceeds that of the brain.
